# Male-pattern hair loss: Comprehensive identification of the associated genes as a basis for understanding pathophysiology

**DOI:** 10.1515/medgen-2023-2003

**Published:** 2023-04-05

**Authors:** Sabrina K. Henne, Markus M. Nöthen, Stefanie Heilmann-Heimbach

**Affiliations:** University Hospital of Bonn & University of Bonn Institute of Human Genetics Bonn Germany; University Hospital of Bonn & University of Bonn Institute of Human Genetics Bonn Germany; University Hospital of Bonn & University of Bonn Institute of Human Genetics Bonn Germany

## Abstract

Male-pattern hair loss (MPHL) is a highly heritable and prevalent condition that is characterized by progressive hair loss from the frontotemporal and vertex scalp. This androgen-dependent hair loss may commence during puberty, and up to 80 % of European men experience some degree of MPHL during their lifetime. Current treatment options for MPHL have limited efficacy, and improved understanding of the underlying biological causes is required to facilitate novel therapeutic approaches. To date, molecular genetic studies have identified 389 associated genomic regions, have implicated numerous genes in these regions, and suggested pathways that are likely to contribute to key pathophysiological mechanisms in MPHL. This review provides an overview of the current status of MPHL genetic research. We discuss the most significant achievements, current challenges, and anticipated developments in the field, as well as their potential to advance our understanding of hair (loss) biology, and to improve hair loss prediction and treatment.

## Introduction

**Figure 1:**
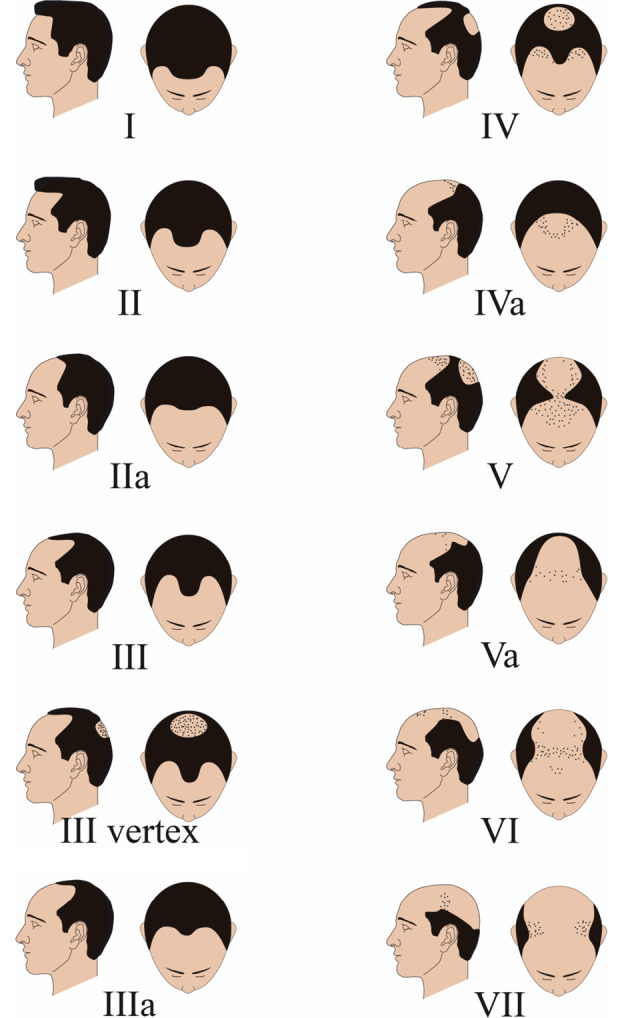
**Clinical classification of MPHL according to the Hamilton-Norwood scale.** MPHL is classified clinically into seven major stages, starting from stage I (no hair loss), a receding of the fronto-temporal hairline (II), regression of the frontal hairline and balding of frontal and vertex scalp areas (III–IV), confluence of the affected areas (V–VI) until only an occipitotemporal hair crown persists (VII) [4]. The less common *a* variant of MPHL progression (IIa, IIIa, IVa, Va) is characterized by recession of the entire anterior hairline without simultaneous development of a bald area on the vertex.

Male pattern hair loss (MPHL), also termed male androgenetic alopecia, is a highly heritable and age-dependent trait. The hair loss may commence during puberty and has a lifetime prevalence of ~80 % in European men [1]. The phenotype is characterized by a distinct pattern of progressive, age-dependent hair loss from the scalp. MPHL typically begins with a bi-temporal recession of the frontal hair line, followed by a thinning of hair in the frontal and vertex scalp areas, which eventually results in complete baldness of the top of the scalp (Figure 1) [2]. In addition to this characteristic pattern of hair loss, the key pathophysiological features of MPHL include a strict androgen-dependency of the phenotype; changes in hair cycle dynamics (i. e. a shorter growth phase [anagen] and a prolonged resting phase [telogen]); and the miniaturization of affected hair follicles (HFs), causing a transition of pigmented terminal hairs to unpigmented vellus hairs [3].

MPHL – especially when occurring in early adolescence – can exert profound negative effects on quality of life [5]. Current therapeutic options for MPHL are limited. At writing, only two U.S. Food and Drug Administration-approved drugs are available, i. e. oral finasteride and topical minoxidil. These vary in terms of efficacy, are non-curative, and can induce severe side effects [3], [6]. The development of novel therapeutic approaches to MPHL will require an improved understanding of the underlying biological causes. This in turn requires the elucidation of the underlying genetic basis. To date, molecular genetic studies of MPHL have identified 389 risk loci, and have led to the identification of pathobiologically relevant genes and pathways for MPHL. This review provides an overview of the key findings, current challenges, and anticipated developments in the field of MPHL genetic research. We also discuss the potential of these developments in terms of advancing our understanding of hair (loss) biology, facilitating drug target identification, and developing new therapies, and in elucidating MPHL pathobiology and its shared biological basis with other traits and diseases.

## MPHL – a highly heritable and polygenic trait

The familial occurrence of MPHL is well documented [7]. While in early research, the pattern of familial occurrence was thought to be compatible with a simple Mendelian mode of inheritance [8], later authors proposed a polygenic model involving a multitude of genes [9], [10] and this hypothesis is now considered proven. Two early twin studies estimated the heritability (h^2^) of MPHL as 0.81 (95 % confidence interval [CI] 0.77–0.85) in early-onset MPHL families (25–36 years), and 0.79 (95 % CI 0.4–0.85) in elderly males (> 70 years), respectively [11], [12]. However, the true heritability may be even higher, since misclassifications of MPHL-severity (e. g. through classification by self-assessment) would lead to an underestimation of MPHL heritability [3]. The strongly heritable and polygenic nature of MPHL was confirmed in a pedigree-based h^2^ analysis in pairs of first-degree relatives from the UK Biobank (UKB) cohort, which generated an h^2^ estimate of 0.62 [13]. The same study found that approximately 60 % of the genetic contribution to MPHL variance was captured by common single nucleotide polymorphisms (SNPs) (h^2^_SNP_ = 0.393), and that the additional inclusion of rare SNPs (1.5x10^–5^ < minor allele frequency [MAF] ≤ 1x10^–3^) had only minor effects on h^2^ estimates (h^2^_SNP_ = 0.415). Of note, pedigree-based h^2^ estimates were slightly lower for father-son correlations compared to those for brother-brother pairs. This may in part reflect the X-chromosome contribution, since fathers and sons do not share X-chromosomal risk factors by descent. One other potential explanation for this observation is that during childhood, brothers share a common environment. However, the issue of whether – and if so to what extent – environmental factors (e. g. smoking, alcohol consumption) are implicated in MPHL development remains a matter of debate [14]–[20].

## Findings from 25 years of gene identification efforts

Since 1998, multiple molecular genetic studies of MPHL have been performed to identify causal genes and pathways. Early MPHL genetic studies were limited to the investigation of only a few, or even single, genes, and generated limited insights into the genetic basis of MPHL. One important caveat here, however, is that even negative findings could not exclude a genuine genetic contribution for the respective gene, since the investigated sample sizes were limited.

A key exception is the X-linked androgen receptor gene (*AR*), which became the focus of a number of studies due to the strict androgen dependency of the MPHL phenotype [21]–[29]. Although these studies generated contradictory results in terms of the associated variants and the most likely causal gene in the region, the genomic region surrounding the *AR* is without question the most strongly associated region for MPHL in the human genome. However, unequivocal identification of the precise causative variants at the X-chromosomal *AR/EDA2R* locus has not yet been achieved, and neither *AR* nor the neighbouring *EDA2R* have been confirmed as the causal gene.

**Figure 2:**
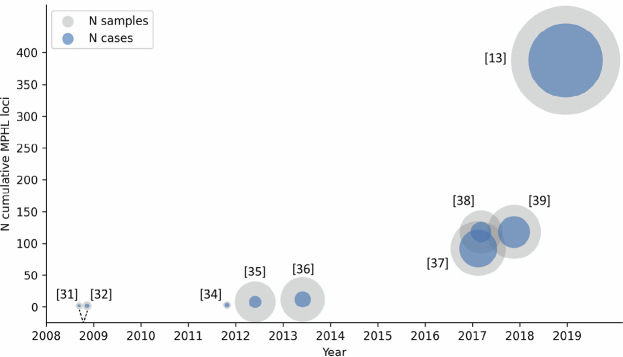
**MPHL susceptibility loci identified via GWAS of individuals of European descent between 2008 and 2022.** MPHL genetic studies are represented by circles. The number of loci denotes the cumulative number of independent MPHL susceptibility loci. The number of individuals and the number of cases in the cohort are reflected by the sizes of the grey and blue circles, respectively. Reference numbers are given in square brackets.

Following these investigations, no notable progress in the identification of additional genetic risk factors was then made until the publication of the first two genome-wide association studies (GWAS) of MPHL in 2008. Since then, GWAS have been performed in increasingly large cohorts, and have generated substantial insights into the role of common genetic variants in MPHL susceptibility (Figure 2). At writing, the GWAS Catalog [30] lists a total of 13 studies under the terms “*androgenetic alopecia”* or “*balding measurement”*, 10 of which report data on MPHL (Table 1). These studies can be broadly categorized into single cohort GWAS, international meta-analyses, and population-based GWAS. The number of reported association signals per study strongly correlates with sample size, and ranges from two independent risk variants in the first published GWAS studies [31], [32] to 622 independent risk variants in the largest GWAS to date [13]. While the individual studies used varying definitions to classify independent risk loci, collapsing of the reported association signals into independent risk loci based on the FUMA tool [33] results in a total of 389 known risk loci for MPHL. Together, these loci explain ~39 % of the observed phenotypic variance in MPHL [13].

The vast majority of these loci are located on the autosomes, and are therefore equally likely to have been inherited from the maternal or the paternal side. Six risk loci, however, are located on the maternally inherited X-chromosome. These include the *AR*/*EDA2R* locus, which has been confirmed as the most strongly associated genomic locus across all studies [13], [31], [32], [34]–[39]. The available data suggest that maternal heredity plays a greater role in the inheritance of MPHL risk than paternal heredity. However, no studies have yet been performed to investigate the existence of paternally inherited Y-chromosomal risk factors.

## From association signals to candidate genes and disease mechanisms

**Figure 3:**
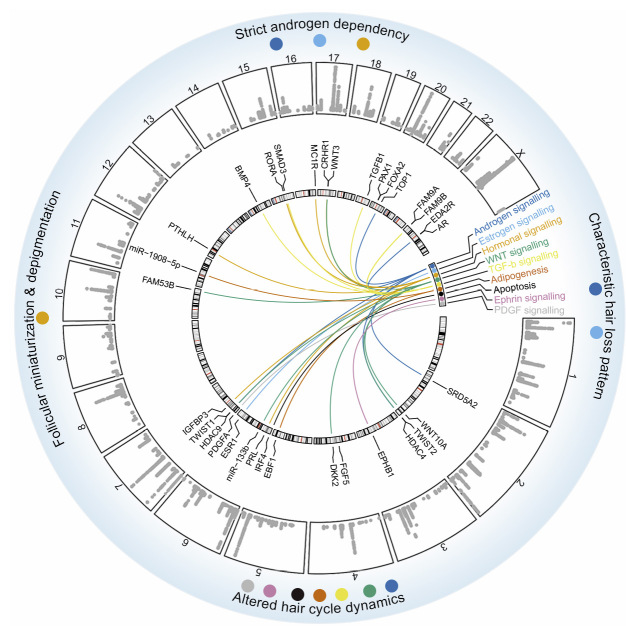
**From genetic association finding to an understanding of MPHL pathobiology.** GWAS have identified 389 independent risk loci and implicated numerous candidate genes and pathways. In some cases, assumptions can be made as to how these genes and pathways contribute to the key pathophysiological features that result in the characteristic MPHL phenotype. Inside to outside: Genomic location (chromosome 1–chromosome X) of candidate genes and assignment to pathways; Manhattan plot of genome-wide significant MPHL SNPs [13] (Y-Axis corresponds to 7–150-log10(P)); key pathophysiological features of MPHL. Colour coding represents the assignment of genes to pathways and their contribution to MPHL key pathophysiological features.

The majority of MPHL risk variants are located in non-coding – and mostly intergenic – regions of the genome. A reasonable assumption is that these variants exert regulatory effects on the expression of relevant genes, e. g. through changes in transcription factor binding sites or enhancer/promoter interaction [41]. While in early GWAS, the proximity of a gene to an association signal was considered the strongest indicator of disease relevance, a number of bioinformatic tools have since been developed that prioritize disease genes and pathways from GWAS data using multiple lines of evidence [33], [42]. The application of these tools to MPHL genetic data has resulted in the identification of a number of interesting genes and pathways (Figure 3). These include the genes encoding i) steroid-5-α-reductase type II (*SRD5A2*), which is the only known therapeutic target for MPHL; ii) *WNT10A* and fibroblast growth factor 5 (*FGF5*), which are known regulators of hair cycling and hair growth, respectively; iii) interferon regulatory factor 4 (*IRF4*), which has previously been reported in connection with hair pigmentation; iv) the transcriptional regulators early B cell factor 1 (*EBF1*), *TWIST1*, and *TWIST2*; and v) other genes involved in the WNT signalling pathway (*DKK2*–Dickkopf 2, *FZD10*–Frizzled class receptor 10, *FAM53B*) or androgen signalling (e. g. *TOP1*, *FAM9B*, *HDAC4*, and *HDAC9*) [3], [38]. Pathway-based and gene-set enrichment analyses have underlined the importance of androgen- and WNT-signalling in MPHL pathogenesis, and have also yielded evidence for a number of other hormonal pathways (e. g. oestrogen- and melatonin signalling, reviewed elsewhere [43]), as well as pathways involved in skin- and epidermal development, apoptosis, and adipogenesis, and immunological processes [13], [38], [39]. Besides indicating the relevance of cells and processes within the HF per se, the latter implicate the perifollicular environment, such as the resident immune cells and adipocytes of the scalp [38].

In studies of MPHL risk variants, regulatory effects on candidate gene expression have been reported at four GWAS loci (1p12–*FAM46C*; 2q35–*WNT10A*; 15q22.2–*RORA*; 21q22.1–*RCAN1*) [44], [45]. Among these, the most notable regulatory effect was found for the MPHL risk allele rs7349332-T at 2q35, which resulted in reduced *WNT10A* expression in human HFs [36]. A follow-up study to investigate the underlying molecular mechanism at this locus identified a nearby binding site for the transcription factor EBF1, whose encoding gene is located at a second MPHL risk locus at 5q33.3. The study demonstrated that EBF1 activates the *WNT10A* promoter, and that this interaction is impacted by the allelic expression of the 2q35 MPHL risk variant. These findings indicate a functional interaction between genes at two independent MPHL risk loci, and suggest that the 2q35 MPHL risk allele results in decreased *WNT10A* promoter activation via EBF1 and a resultant lowering of *WNT10A* expression, which eventually impacts HF cycling [46].

**Table 1:** **Overview of GWAS, number of reported independent associations (P < 5x10^–8^), and key findings to date.** When available, the number of samples and cases is shown.

**Authors**	**Year**	**Study type**	**N samples (cases)**	**N associations** **(P < 5x10^–8^)**	**Key findings**
Hillmer et al. [31]	2008	GWAS	643 (296)	2	Confirmation of the* AR/EDA2R* locus, novel risk locus on chr20p11.22 (concurrent with [32])
Richards et al. [32]	2008	GWAS	1125 (578)	2	Confirmation of the* AR/EDA2R* locus, novel risk locus on chr20p11.22 (concurrent with [31])
Brockschmidt et al. [34]	2011	GWAS	1198 (581)	3	Novel risk locus on chr7p21.1, implicating *HDAC9*
Li et al. [35]	2012	Meta-analysis	12,806 (3,891)	8	1^st^ international meta-analysis of the MAAN consortium. Identification of 5 novel autosomal risk loci, unexpected association with Parkinson’s disease
Heilmann et al. [36]	2013	Candidate variant analysis	5,420 (2,759)	4	First genetic evidence for an involvement of WNT-signalling (*WNT10A*, 2q35) in MPHL
Pickrell et al. [40]	2016	GWAS	17,500 (9,009)	49	Evidence for pleiotropic effects of MPHL risk variants on lower age at menarche
Hagenaars et al. [37]	2017	Population-based GWAS	52,874 (36,150)	287	1st GWAS based on data from the UK Biobank, development of a polygenic prediction model
Heilmann-Heimbach et al. [38]	2017	Meta-analysis	22,518 (10,846)	63	2nd MAAN analysis, implication of numerous candidate genes and pathways, evidence for shared genetic basis between MPHL and other traits and illnesses
Pirastu et al. [39]	2017	Population-based GWAS	43,590 (25,662)	71	Implication of plausible additional candidate genes and pathways (e. g. TGF-beta, apoptosis signalling)
Yap et al. [13]	2018	Population-based GWAS	205,327	622	Largest GWAS to date

While the underlying genes and precise biological mechanisms at the majority of loci remain elusive, for some loci and genes, hypotheses can be formulated as to how they will contribute to key pathophysiological processes in MPHL. These include deregulation of the hair growth cycle (*FGF5*, *EBF1*, *DKK2*, adipogenesis, WNT-signalling), mediation of androgen sensitivity (*SRD5A2*, AR-signalling, melatonin signalling), and the conversion of pigmented terminal into non-pigmented vellus-like hairs (*IRF4*) (Figure 3).

## Epidemiological associations and genetic pleiotropy – placing MPHL into a wider medical context

Epidemiological studies have reported associations between MPHL and a number of medical conditions and anthropometric indices, including cardiovascular disease [47]–[54], type 2 diabetes [18], [53], [55]–[58], BMI [18], [58], [59], metabolic syndrome [58], [60]–[63], benign prostate hyperplasia [64], [65] and prostate cancer [66], [67]. The observed epidemiological associations suggest an overlap in pathobiology, which may be explained at least in part by shared genetic factors. Using GWAS data, genome-wide significant correlations have been detected for MPHL and metrics of early puberty [13], [40], bone mineral density (↑) [13], HOMA-B levels (↑) [13] and human body height (↓) [39]. Since early puberty is associated with childhood adiposity and increased BMI in adulthood [68], and higher HOMA-B levels (a measure of pancreatic β-cell function), are associated with an increased BMI [69], these genetic correlations may partly explain the reported epidemiological association between MPHL and metabolic syndrome, and between MPHL and BMI [13], [39]. At individual loci, overlapping associations have been reported for prostate cancer and cardiovascular traits, although these did not reflect in a significant genome-wide genetic correlation [13], [18], [37], [40].

A study in the Icelandic population found a pleiotropic variant for MPHL and reduced female reproductive rate on ch.17q21.31 [35], [70]. Notably, polycystic ovary syndrome (PCOS), the most common cause of reduced fertility in women, is frequently observed in the female relatives of MPHL-affected men [71], [72], and a Mendelian randomization study found that MPHL risk variants are associated with increased PCOS risk [73]. However, a genome-wide genetic correlation analysis found no significant correlation between the two traits [73].

At single loci, overlapping genetic associations with MPHL have also been reported for Parkinson’s disease (PD) and amyotrophic lateral sclerosis (ALS) [35]. These findings were supported by subsequent epidemiological investigations, which revealed an increased risk for PD (1.3-fold increased risk; 95 % CI 1.06–1.55 [35]) and ALS (2.7-fold increased risk; 95 % CI 1.23–6.31 [74]) in MPHL-affected compared to unaffected men. However, no genome-wide genetic correlations were observed.

More recently, research has suggested that MPHL is a possible risk factor for severe COVID-19, as based on the high incidence of severe MPHL in hospitalized COVID-19 patients [75]. However, subsequent epidemiological and genetic correlation analyses did not confirm this epidemiological association [76]–[78], and no general genetic correlation was identified [76], [79], although pathway-based polygenic risk score analyses suggested a potential limited biological overlap between the traits [76].

In many cases, steroid hormone or androgen signalling may act as the common biological mechanism for MPHL and the associated traits. Androgen-mediated neurotoxicity has been discussed as a possible mechanism in the pathogenesis of PD, and could also partially explain the association with ALS [80], [81]. In women, PCOS is promoted by elevated androgen levels [82]. Androgen- and oestrogen-signalling, together with other MPHL-associated hormonal pathways, have also been implicated in skeletal development and bone homeostasis [83], [84].

Future studies are required to determine the extent to which these pleiotropies can indeed be explained by common hormonal pathways, and to identify other contributory biological signalling pathways. A deeper understanding of pleiotropies and the underlying biological mechanisms will have important implications for risk prediction and drug therapy, both in terms of drug repurposing and the prediction of side effects.

## To lose or not to lose – MPHL risk predicted from genetic data

Although several MPHL prediction models have been developed, to date, these have yielded only limited predictive value at the individual level [37], [85]–[87]. The most recent model was based on 160,267 male participants from the UKB, with testing having been performed in an additional 26,177 male UKB participants as well as in an independent cohort of 991 males enriched for early-onset (< 40 years) severe MPHL cases [87]. When predicting MPHL with differing degrees of severity, the model achieved prediction accuracies ranging from 0.60 – 0.73, as based on the area under the curve (AUC), with higher degrees of severity being predicted with greater accuracy. A prediction accuracy of 0.83 was achieved when predicting the presence of any hair loss in the early-onset (< 40 years) cohort. Notably, the strongest predictor was age, followed by rs12558842, which is located ~280kb upstream of *AR* [87]. Although the overall prediction accuracy and reliability of the model have improved substantially over time, the accuracy of individual predictions remains limited, in particular for mild and medium levels of balding (Hamilton-Norwood-scale < VI). Prediction may therefore be too crude for reliable estimates of metrics such as an individual’s age of onset or their rate of MPHL progression. While such models could now be expanded due to the discovery of novel risk loci and variants [13], further investigations of additional genetic risk factors and potential environmental factors, and the use of large independent data sets, will also be required to improve current MPHL prediction models. Moreover, existing prediction models are based on Euro-centric GWAS, which will likely hamper their application in other ethnicities [88]. Once successfully established, MPHL prediction models may be valuable in a (wider) medical and forensic context. Prediction could also help to determine the feasibility and appropriate time for drug interventions and hair transplantations, for which therapeutic efficiency is strongly dependent on the clinical stage and the rate of disease progression [6], [89].

## MPHL genetic studies in non-European ethnicities

Epidemiological research indicates that MPHL manifests slightly differently in non-European ethnicities in terms of prevalence, onset, and pattern of progression [1], [90]–[92]. However, most genetic studies to date have focused on Europeans, with genetic studies in non-European populations having been limited to one GWAS of combined female-pattern hair loss (FPHL) and MPHL in admixed Latin Americans [93], two replication studies in Asian populations [94], [95], and one small-scale autosomal GWAS of MPHL in a cohort from Korea [95]. The Latin American GWAS identified associations at the *AR/EDA2R* locus, with the most significant variant rs4258142 having been previously described in Europeans, and rs2814331 at the 10q23.2 locus implicating *GRID1* as a novel candidate gene [93]. However, since MPHL and FPHL were jointly analysed, it is unclear whether this association was attributable to MPHL. The Korean-based GWAS identified 13 suggestive (P < 10^–5^) risk loci (1p21.1, 4q22.3, 4q35.1, 5p15.33, 7q31.31, 9q21.31, 9q21.33, 10p11.21, 10q23.31, 11q21, 11q25, 12p11.21, 18p11.21), one of which (9q21.31) had been described in a previous European-based GWAS. The authors also investigated SNPs with a previously reported association with MPHL in European-based GWAS. The strongest nominally significant (P < 0.05) associations were found at 20p11 [95], as in a previous replication attempt in the Chinese Han population [94].

Notably, the association at the *AR/EDA2R* locus could not be replicated in the Asian studies, since previously implicated SNPs at the locus were monomorphic or nearly monomorphic (MAFs < 1 %) for the European MPHL risk alleles in the Korean and Chinese Han populations [94], [95]. This may either point to a different allelic architecture and/or haplotype constellation between European and Asian populations at the *AR/EDA2R* locus or a general genetic heterogeneity for MPHL between these populations. Independent analyses of the X chromosome are therefore required to clarify the role of variants at the *AR/EDA2R* locus in Asian populations. While the association findings in non-European populations require replication in larger cohorts, they suggest the presence of distinct risk loci in non-European ethnicities. Further studies in diverse populations are required to shed light on genetic determinants in other ethnicities. This could facilitate further exploration of MPHL pathobiology and the identification of mechanisms that contribute to ethnic differences, such as age of onset and balding severity, in specific subpopulations of androgen-sensitive scalp HFs.

### Overlap between MPHL and female-pattern hair loss

The present review has focused on the advances and challenges of genetic research into MPHL, which raises the question of the extent to which these findings also apply to FPHL. The two conditions share key characteristics, such as high prevalence, the age-dependent and progressive nature of the hair loss, and key pathophysiological characteristics, such as follicular miniaturization and deregulation of hair cycle dynamics [2], [96]. However, research suggests that MPHL and FPHL are distinct entities with differing pathogeneses [97], and the relevance of genetic factors and the role of androgens in FPHL is less clear than is the case for MPHL [96], [97]. While previous authors have suggested that FPHL has a heritable component [98], [99], the evidence is inconclusive [97]. Genetic studies of FPHL have largely focused on specific genes related to steroid hormone metabolism and on risk variants and loci previously identified in MPHL. Together, these studies have implicated a number of candidate genes (*CYP19A1*, *ESR2, PPARGC1A, ABCC4, CYP11B2, FSHB*) and risk loci (*AR/EDA2R,* 20p11) [97], [100] – [111]*.* To date, no GWAS of FPHL has yet been published. GWAS in larger FPHL cohorts are required to improve estimation of the contribution of genetic factors, and to generate novel insights into the underlying genetic architecture.

## Challenges and perspectives in MPHL genetic research

MPHL is widely misconceived as a phenotype of mainly cosmetic and psychological importance. However, given its high prevalence, simple clinical classification, strict hormone dependency, sex-limited expression, and highly polygenic basis, MPHL is an interesting model phenotype for the study of traits with similar features.

Available GWAS of MPHL have identified associations with thousands of common variants at 389 loci, and have yielded unprecedented insights into the genetic and biological basis of what is the most common form of hair loss in men. Together, these genetic findings explain ~39 % of the observed phenotypic variance of MPHL in the white British population [13], [39], mixed European populations [38], and Latin American populations [93]. Research has confirmed that MPHL has a strong genetic contribution, with SNP-based h^2^ estimates of 60–70 % [13]. However, research has yet to demonstrate the existence of genetic factors that may bridge the gap between the SNP-based and the twin-study h^2^ estimates of MPHL heritability. While a general hypothesis is that rare genetic variants contribute to complex disease heritability, their estimated contribution to MPHL is minimal [13], and to date, exome-based phenome-wide association studies (PheWAS) have identified no associations between rare variants and MPHL [112], [113]. Despite the fact that rare variants are unlikely to contribute in general to MPHL heritability, the possibility that they play a role in some individuals and families cannot be excluded. However, in contrast to common variants, few data are available concerning the contribution of individual rare variants (MAF < 1 %) to MPHL in phenotypically well characterized or family-based cohorts. Each of these rare genetic findings will add important information on the genotype-phenotype correlation, and may provide important (new) insights into MPHL pathobiology. Future genetic studies are warranted to decipher the full allelic spectrum of MPHL, and these should include larger cohorts and the investigation of the full spectrum (common and rare variants) of sequence variation through whole-exome or whole-genome sequencing. Comprehensive understanding of MPHL phenotypic variance in the population will require the investigation of the genetic – and potentially also the non-genetic factors – that impact hair loss progression. This in turn will require longitudinal studies in large cohorts with extensive phenotypic and comprehensive genetic data. Furthermore, future systematic investigations into the genetic basis of MPHL both within different ethnicities and across different ethnicities will yield further insights into MPHL pathobiology, and will enable the generation of improved and trans-ethnic prediction models.

In addition to the further dissection of the genetic basis of MPHL, another important and likely even greater challenge will be the functional annotation and biological interpretation of current and future genetic findings. Although several plausible genes and pathways have been identified, the detection of the causal genes and pathways, and our understanding of the precise underlying biological mechanisms, remain in their infancy. Despite the fact that the functional annotation of non-coding regions in the genome is becoming increasingly detailed [114], further experiments are required to accurately reflect the full spectrum of regulatory interactions, which are often tissue-, cell type-, or context-specific. This is also true for MPHL, for which only a limited number of human data sets are available for the identification of causal genes and pathways and the investigation of the molecular mechanisms that underlie the characteristic hair loss.

Systematic follow-up of all association findings for MPHL is warranted, and this is likely to involve two key approaches. First, the development of sophisticated bioinformatic analysis strategies will probably be required to allow the performance of pathway- and network-based analyses that elucidate the molecular mechanisms at individual and across loci. Second, (single-cell) expression profiling of genes and signalling pathways will probably be necessary in order to determine the relevant HF and/or perifollicular compartments in which known, and as yet unidentified, pathways interact to cause the characteristic androgen-dependent hair loss.

As discussed, current therapeutic options for MPHL are suboptimal, and knowledge of MPHL relevant genes and pathways will facilitate the development of further (more effective) therapies. In a first step towards this, work by Pirastu *et al.* and our group showed that 45 MPHL candidate genes were druggable targets [39, own unpublished data]. These findings are encouraging, since drugs based on targets or mechanisms for which genetic evidence is available are more likely to succeed in clinical trials [115], [116]. Furthermore, due to the highly polygenic nature of MPHL, genetically guided drug research and patient stratification may eventually lead to the accurate prediction of therapeutic success and adverse side effects, thus enabling precision medicine approaches.
